# Hybrid Ni@ZnO@ZnS‐Microalgae for Circular Economy: A Smart Route to the Efficient Integration of Solar Photocatalytic Water Decontamination and Bioethanol Production

**DOI:** 10.1002/advs.201902447

**Published:** 2019-12-12

**Authors:** Albert Serrà, Raül Artal, Jaume García‐Amorós, Borja Sepúlveda, Elvira Gómez, Josep Nogués, Laetitia Philippe

**Affiliations:** ^1^ Empa Swiss Federal Laboratories for Materials Science and Technology Laboratory for Mechanics of Materials and Nanostructures Feuerwerkerstrasse 39 CH‐3602 Thun Switzerland; ^2^ Grup d'Electrodeposició de Capes Primes i Nanoestructures (GE‐CPN) Departament de Ciència de Materials i Química Física Universitat de Barcelona Martí i Franquès, 1 E‐08028 Barcelona Catalonia Spain; ^3^ Institute of Nanoscience and Nanotechnology (IN2UB) Universitat de Barcelona E‐08028 Barcelona Catalonia Spain; ^4^ Grup de Materials Orgànics Departament de Química Inorgànica i Orgànica Universitat de Barcelona Martí i Franquès, 1 E‐08028 Barcelona Catalonia Spain; ^5^ Catalan Institute of Nanoscience and Nanotechnology (ICN2) CSIC and BIST Campus UAB Bellaterra E‐08193 Barcelona Spain; ^6^ ICREA Pg. Lluís Companys 23 E‐08010 Barcelona Spain

**Keywords:** bioethanol production, biomimetics, biotemplating, photocatalysis, water decontamination

## Abstract

Water remediation and development of carbon‐neutral fuels are a priority for the evermore industrialized society. The answer to these challenges should be simple, sustainable, and inexpensive. Thus, biomimetic‐inspired circular and holistic processes combing water remediation and biofuel production can be an appealing concept to deal with these global issues. A simple circular approach using helical *Spirulina platensis* microalgae as biotemplates to synthesize Ni@ZnO@ZnS photocatalysts for efficient solar water decontamination and bioethanol production during the recycling process is presented. Under solar irradiation, the Ni@ZnO@ZnS‐*Spirulina* photocatalyst exhibits enhanced activity (mineralization efficiency >99%) with minimal photocorrosion and excellent reusability. At the end of its effective lifetime for water remediation, the microalgae skeleton (mainly glycogen and glucose) of the photocatalyst is recycled to directly produce bioethanol by simultaneous saccharification and fermentation process. An outstanding ethanol yield of 0.4 L kg^−1^, which is similar to the highest yield obtained from oxygenic photosynthetic microorganisms, is obtained. Thus, the entire process allows effective solar photocatalytic water remediation and bioethanol production at room temperature using simple and easily scalable procedures that simultaneously fixes carbon dioxide, thereby constituting a zero‐carbon‐emission circular process.

## Graphical abstract

A holistic, green, circular, scalable, and inexpensive process that enables integrating CO_2_ fixation, water decontamination, and bioethanol production is developed. Microalgae are used for CO_2_ fixation and as skeletons to synthesize effective visible‐light photocatalysts. At the end of the photocatalyst lifetime, hybrid structures are recycled to produce bioethanol by a simultaneous saccharification and fermentation of the algae biomass.

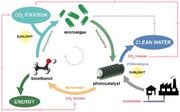

## 1 Introduction

Unsustainable societal development and rapid global industrialization have caused a high stress on water and energy resources, particularly drinking water and carbon fuels. Consequently, efficient water decontamination and replacement of current dense energy carriers with removable alternatives are extremely important in mitigating the highly undesirable consequences of unsustainable growth on health, economy, and enviçronment.^1^ In this context, the development of circular and holistic processes mimicking the biological life cycle, where every end is a new beginning, is crucial to achieving a more sustainable future.^2^

The design and implementation of competitive circular processes must integrate the biomimetic rationale to satisfy four essential criteria: simplicity, efficiency, cost‐effectiveness, and material availability. Sunlight, as the main engine of most of the biological process, offers the key advantages of being a virtually inexhaustible and clean source of energy. Consequently, diverse processes for the conversion of solar radiation to chemical fuels, electricity, or heat have generated enormous interest as a solution to the issue of global energy deficiency and environmental pollution.^3^ In particular, ongoing energy research focuses on the use of new materials to improve the photocatalytic efficiency in the visible‐light range and development of artificial photosynthesis prototypes and strategies to obtain carbon‐neutral fuels. In addition, solar photocatalysis has been intensively studied for water decontamination (i.e., pollutant mineralization).^3^

Semiconductors based on metal oxides (e.g., ZnO, TiO_2_) and sulfides, such as CdS, have demonstrated great potential as photocatalysts for different processes. However, their performance remains suboptimal because of i) limited visible‐light photocatalytic activity; ii) insufficient efficiency to trap and harvest sunlight; iii) limited photocorrosion resistance, and/or iv) high potential ecotoxicological effects on aquatic biota.^4^ Hence, different approaches (e.g., doping, complex architectures, or heterojunctions) are being pursued to mitigate their shortcomings. In particular, current research on photocatalyst synthesis also focuses on the use of bioinspired and biomimetic architectures to improve light trapping and harvesting capabilities. Nevertheless, the combined overall performance of current photocatalysts in the visible region of the electromagnetic spectrum remains exceedingly low for commercial exploitation. Therefore, the development of efficient, green, and sustainable photocatalysts that fulfill all the exigent performance requirements is still necessary.^4^, ^5^

The current pursuit of sustainable solutions to energy and environmental issues is not only limited to the development of efficient photocatalysts. Today, researchers also devote significant effort on the replacement of fossil fuels with bioenergy, especially carbon‐neutral biofuels.^6^ Bioethanol is viewed as one of the best alternatives because of its similarity to petrol. However, the existing technology is unsustainable for the large‐scale production of bioethanol. Nevertheless, photosynthetic microorganisms such as microalgae can produce large amounts of biomass by consuming only sunlight, carbon dioxide, and some nutrients, serving as a high‐carbon source. In this context, microalgae, specially cyanobacteria, are one of the most promising options for bioethanol (third‐generation biofuel) production owing to their characteristics such as high biomass productivity, high capacity to fixate carbon dioxide, and possibility of growth in seawater (salt tolerance).^6^ Current efforts are concentrated on enhancing bioethanol yield by developing diverse strategies to promote the extraction of intracellular carbohydrates. Interestingly, the latter, mainly glycogen, can be easily extracted from some cyanobacteria, such as *Spirulina platensis*, without complex pretreatments due to their extremely brittle membrane; thus, these cyanobacteria are excellent candidates for bioethanol production.^6^

In the present study, we developed a novel hybrid structure consisting of *Spirulina plantensis* microalgae plated by Ni@ZnO@ZnS shells for a high‐yield circular process merging water decontamination and biofuel production. To close the cycle, the clean water and the CO_2_ produced in this diverse process are used to cultivate new algae.

**Scheme 1:**
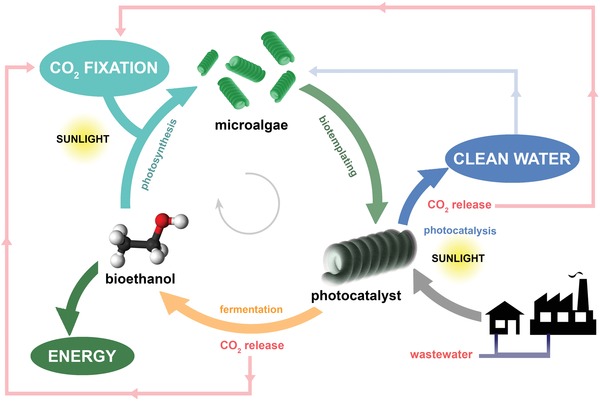
Schematic illustration of the circular process using microalgae (*Spirulina plantensis*) for water mineralization and bioethanol production.

The hybrid Ni@ZnO@ZnS‐*Spirulina* has enabled, for the first time, the integration of i) highly efficient biomimetic solar photocatalysis for water decontamination, ii) effective bioethanol production by the *Spirulina* recycled at the end of the photocatalyst's lifetime, and iii) fixation of carbon dioxide (**Scheme** 1). The Ni@ZnO@ZnS‐*Spirulina* photocatalysts exhibited excellent solar photocatalytic performance for the mineralization of methylene blue (MB) with minimal photocorrosion activity. Additionally, the recycled photocatalyst reached a conversion efficiency of ≈96% for bioethanol production (0.4 L of bioethanol per kg of dry photocatalyst) after saccharification and fermentation. Therefore, an outstanding circular process for integrated CO_2_ fixation, water decontamination, and bioethanol production has been demonstrated.

## 2 Results and Discussion

### 2.1 Preparation of the Hybrid Ni@ZnO@ZnS‐Microalgae Photocatalyst

The photocatalytically active microalgae hybrid is composed of *Spirulina plantensis* coated with an onion‐like Ni@ZnO@ZnS shell. The *Spirulina* acts as the structural support of the magnetic and photocatalytic layers and as biomass reservoir for efficient bioethanol production. Note that the Ni layer in the structure has a dual role, i) providing mechanical strength to the fragile *Spirulina* and serving as a suitable surface for the growth of ZnO and ii) given its magnetic properties, it enables the magnetic manipulation of the photocatalyst to transport it to the reaction site and to easily recover it after its lifetime. Finally, the ZnO/ZnS heterostructure coating constitutes a highly efficient and stable photocatalytic layer for water decontamination by solar photocatalysis. This multifunctional microstructure can be fabricated following a simple, cost‐effective, and scalable process, as follows (see Experimental Section in the Supporting Information for details):

#### 2.1.1 Optimum Spirulina Cultivation

Prior to biotemplating, the microalgae were cultivated for 5 days in a nitrate‐deficient (4 × 10^−3^ m NaNO_3_) algal culture medium to increase the glycogen content, and consequently the bioethanol production yield, in the subsequent simultaneous saccharification and fermentation.[qv: 6e,f,7] Under these conditions, the glycogen content (67.4 ± 2.1% w/w of dry the microalgae weight) is ≈2.5 times higher than that of the microalgae cultivated in conventional microalgae media (30 × 10^−3^ m NaNO_3_), whereas the glucose content is not influenced by the nitrate ion concentration (Table S1, Supporting Information). In addition, the biochemical composition remains virtually constant even after two weeks of fixation.

#### 2.1.2 Biotemplating Process

**Figure 1:**
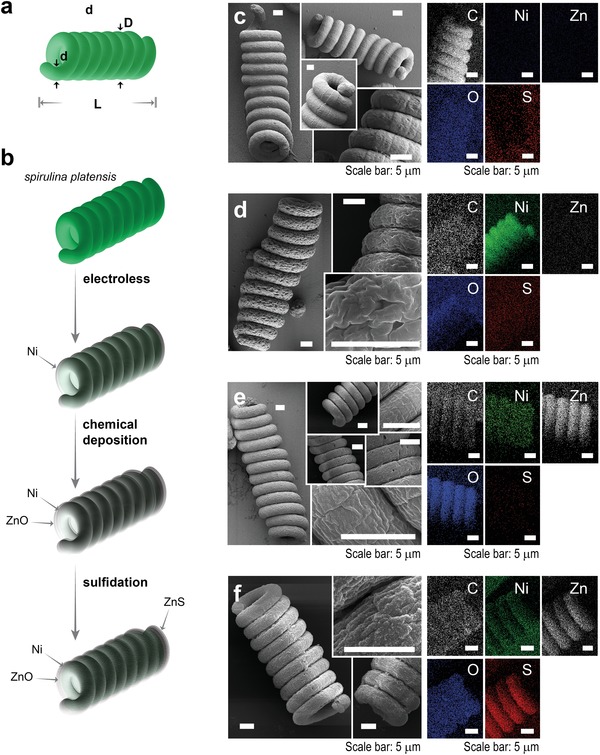
a) Geometric parameters of *Spirulina*: wire diameter (*d*), helix diameter (*D*), and length (*L*). b) Schematic representation of the biotemplating process. Field emission‐scanning electron microscope micrographs and EDX mapping of c) fixed *Spirulina* platensis, d) Ni‐*Spirulina*, e) Ni@ZnO‐Spirulina, and f) Ni@ZnO@ZnS‐*Spirulina*.

Biotemplating (**Figure** 1; Scheme S1, Supporting Information) involves three steps: i) Ni electroless plating, ii) ZnO chemical deposition, and iii) ZnS sulfidation (see Methods for details).^8^ The process is initiated by the fixation of *Spirulina plantensis* by glutaraldehyde to maintain the initial shape and prevent microalgae fragmentation. Prior to the Ni electroless deposition, Pd nanoparticles are adsorbed on microalgae surface to act as a nuclei catalyst to promote the Ni deposition. The Pd‐covered *Spirulina* biotemplate (Figure 1c) has the common compact helix structure of *Spirulina plantensis* var lonar consisting of a microhelix ≈3.9 µm in wire diameter, ≈23 µm in helix diameter, and ≈65 µm in length (8–12 turns). Subsequently, the surface of the *Spirulina* is completely covered with a Ni layer (Figure 1d). Note that this Ni layer serves two purposes: i) to provide the structure with magnetic functionality that enables magnetic collection of the hybrid microalgae (Figure S2, Supporting Information) and ii) to allow the deposition of the ZnO semiconductor layer. As can be seen in Figure 1d, Ni is successfully deposited only on the *Spirulina* microhelix surface, which is consequently rough, microporous, and magnetic. The estimated thickness of the Ni shell is ≈ 1.5 µm. The Ni‐*Spirulina* is then completely covered by a thin ZnO layer that fills the Ni micropores, as shown in Figure 1e. Finally, a nanometric ZnS shell is successfully formed by partial sulfidation of the ZnO layer (Figure 1f), resulting in a Ni@ZnO@ZnS‐*Spirulina* photocatalyst with an onion‐like core@shell@shell structure. Sulfidation enhances the surface roughness, which is desirable to increase the effective surface area of a photocatalyst. The average geometrical and structural parameters of the hybrid *Spirulina* are summarized in **Table** 1. In particular, the Brunauer–Emmett–Teller (BET) surface area of Ni@ZnO@ZnS‐*Spirulina* is 79.1 m^2^ g^−1^, which is 1.12 times higher than that of the Ni@ZnO‐Spirulina, suggesting that the sulfidation, indeed, increases the surface roughness. However, it is 0.79 times lower than that of the Ni‐*Spirulina* because of the micropore filling (Figure S3, Supporting Information). Notably, the specific surface area accessible to pollutants and, more importantly, light, plays an essential role in the photocatalytic degradation efficiency by providing more active sites.[qv: 4a,5c]

**Table 1:** Average feature sizes, BET surface areas, and bandgap energies of the different *Spirulina* structures

*Spirulina*	Wire diameter (*d*) [µm]	Helix diameter (*D*) [µm]	Length (*L*) [µm]	BET surface area [m^2^ g^−1^]	Bandgap energy [eV]
Ni	4.5–7.1	25–29	60–70	118.1	–
Ni@ZnO	4.8–7.3	25–30	60–70	70.6	3.21 ± 0.09
Ni@ZnO@ZnS	4.8–7.4	25–30	60–70	79.1	2.85 ± 0.06

The successful growth of Ni, ZnO, and ZnS is confirmed by energy‐dispersive X‐ray (EDX) elemental mappings (Figure 1), X‐ray diffraction (Figure S4, Supporting Information), and X‐ray photoelectron spectroscopy spectra (Figure S5, Supporting Information). In particular, the EDX mapping of each *Spirulina* structure demonstrates that the biotemplating process is consistent, that is, the growth is uniform and homogeneous across the entire surface of microalgae, leading to a multilayered core@shell@shell microstructure.

### 2.2 Basic Optoelectronic Characterization

**Figure 2:**
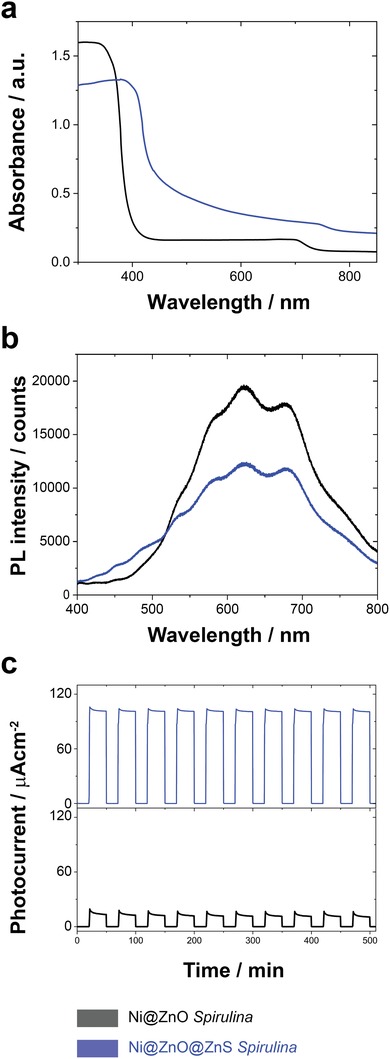
a) UV–vis diffuse reflectance absorption spectra, b) photoluminescence (PL) spectra, and c) transient photocurrent response under UV‐filtered irradiation (λ > 400 nm) of Ni@ZnO‐ and Ni@ZnO@ZnS‐*Spirulina*.

The optoelectronic properties of Ni@ZnO‐ and Ni@ZnO@ZnS‐*Spirulina* were investigated by UV–vis diffuse reflectance, photoluminescence spectroscopies, and photoelectrochemical tests (**Figure** 2). As can be seen in Figure 2a, the Ni@ZnO‐Spirulina exhibits the characteristic spectrum of ZnO, with a fundamental sharp absorption edge rising at ≈390 nm and a very small absorption intensity in the visible domain. However, after sulfidation (Ni@ZnO@ZnS‐*Spirulina*), the absorption band is displaced to the visible domain, which should lead to a stronger photoresponse in this region. In fact, the bandgaps of the Ni@ZnO‐ and Ni@ZnO@ZnS‐*Spirulina* structures, calculated using the Tauc relation (Figure S6, Supporting Information), are 3.21 ± 0.09 and 2.85 ± 0.09 eV, respectively.[qv: 4b,9]

The Ni@ZnO‐*Spirulina* exhibits an intense and broadband photoluminescence spectrum centered at 630 nm, which is indicative of the polycrystalline structure of the ZnO layer with multiple trapped electronic states due to the crystallographic defects. Interestingly, sulfidation substantially reduces the luminescence intensity. This can be attributed to the passivation of some of the crystallographic defects by the mechanical stress generated by the incorporation of S atoms in the lattice during the sulfidation.[qv: 4b,9]

As shown in Figure 2c, reversible, uniform, and stable photocurrent responses were observed for Ni@ZnO‐, and Ni@ZnO@ZnS‐*Spirulina*; during ten on/off cycles (30 s/20 s), the current increased rapidly under UV‐filtered irradiation and recovered quickly when the light was turned off. Additionally, the photocurrent enhancement observed for Ni@ZnO@ZnS‐*Spirulina* (about 6.4 times higher than that of Ni@ZnO‐Spirulina) indicates an improved separation efficiency of the photogenerated electrons and holes, attributed to the synergetic interaction between ZnO and ZnS. This confirms that ZnO@ZnS core@shell photocatalysts have improved photocatalytic properties over pure ZnO photocatalysts.

These optoelectric properties of the Ni@ZnO@ZnS‐*Spirulina* already suggest its high potential for enhanced UV‐filtered photocatalytic response.

### 2.3 Water Decontamination: Photocatalytic Mineralization of Persistent Organic Pollutants (POPs)

The photocatalytic mineralization of POPs is governed by the generation of reactive oxygen species (ROS), mainly hydroxyl radicals (•OH), on the photocatalyst surface. However, oxygen superoxide ions (O_2_^−^) and singlet oxygen (^1^O_2_) can also play an important role; this role drastically depends on the pH. Consequently, prior to evaluating the photomineralization efficiency of ZnO‐based *Spirulina* photocatalysts, the formation of ROS species (Figure S7, Supporting Information) under UV‐filtered simulated sunlight (>400 nm, light intensity of 680 ± 10 lx) at 25 °C was determined by using chemical selective radical quenchers.^4^, ^10^ Specifically, the kinetics and concentration of hydroxyl radicals was investigated using a fluorescein salt as the selective radical quencher, assuming zero order kinetics and equimolar reaction stoichiometry between hydroxyl radicals and fluorescein molecules. As can be seen in Figure S7a,b in the Supporting Information, Ni@ZnO@ZnS‐*Spirulina* exhibited ≈30 times higher production of hydroxyl radicals (0.48 × 10^−6^ m min^−1^) than did Ni@ZnO‐Spirulina (0.016 × 10^−6^ m min^−1^), which suggests that Ni@ZnO@ZnS‐*Spirulina* has high potential in mineralizing POPs. In addition, the formation of oxygen superoxide ions was determined by monitoring the reaction between 2,3‐bis(2‐methoxy‐4‐nitro‐5‐sulfophenyl)‐2*H*‐tetrazolium‐5‐carboxanilide sodium salt (XTT) and oxygen superoxide ions (Figure S7c, Supporting Information) by means of conventional time‐resolved UV–vis spectroscopy. The formation of singlet oxygen was also determined by monitoring the reaction of the anthracene platform of the SOSG reagent with singlet oxygen. In these experiments, Ni@ZnO@ZnS‐*Spirulina* presented a relatively low activity in producing oxygen superoxide ions and singlet oxygen compared to Ni@ZnO‐*Spirulina*. The photolytic formation of ROS from water was negligible. Consequently, a high photomineralization efficiency of POPs is expected for Ni@ZnO@ZnS‐Spirulina under UV‐filtered simulated sunlight due to the high production of hydroxyl radicals.

**Figure 3:**
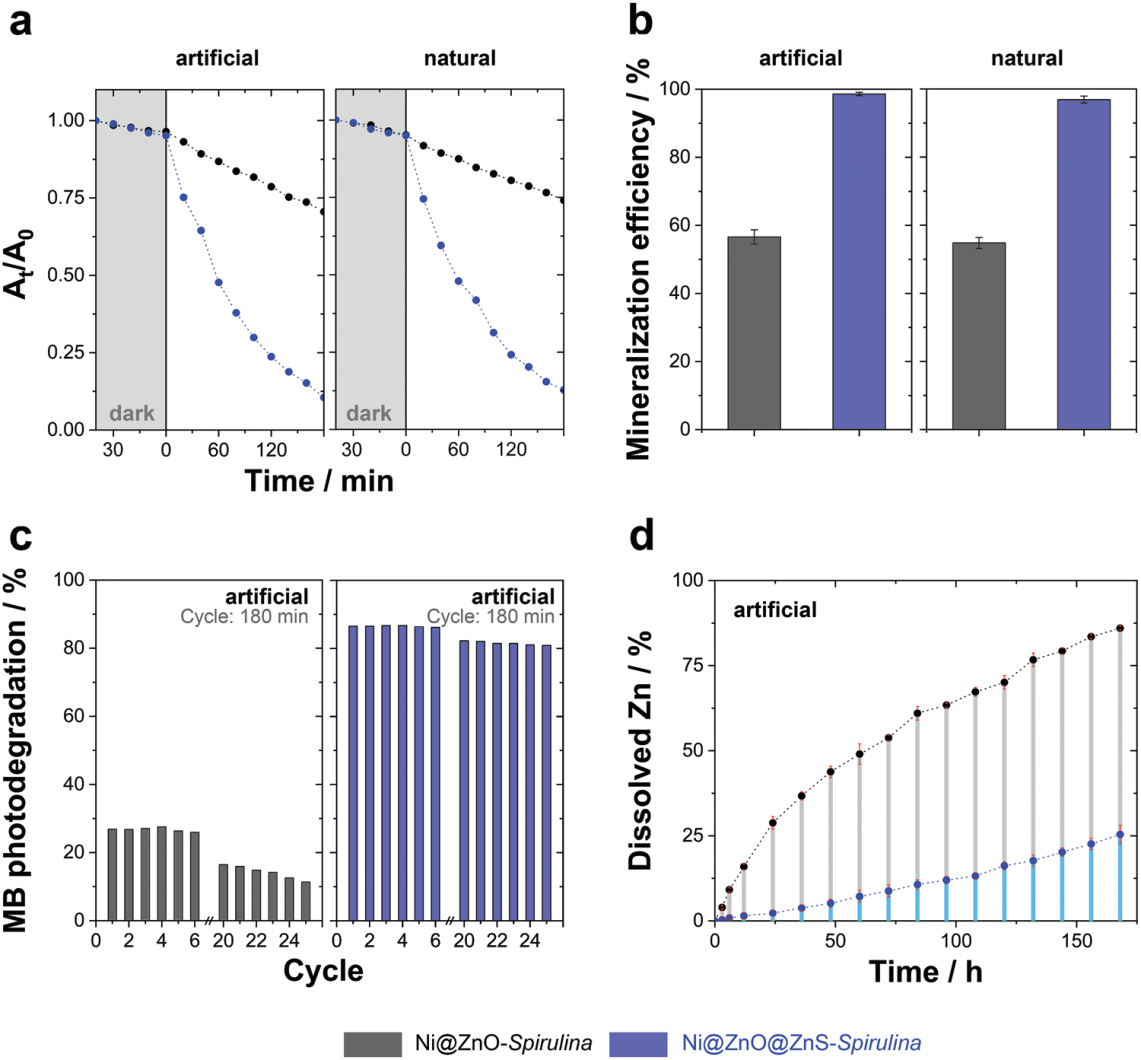
a) Photodegradation and b) mineralization efficiency of MB (10 ppm in algae culture medium) under artificial and natural UV‐filtered sunlight irradiation of the Ni@ZnO‐ and Ni@ZnO@ZnS‐*Spirulina* photocatalysts. c) MB photodegradation efficiency of the photocatalysts during 25 consecutive recycling cycles under artificial UV‐filtered sunlight irradiation. d) Time‐dependent dissolution of Zn(II) from the photocatalysts under artificial UV‐filtered sunlight irradiation. Photocatalyst dosage = 0.5 mg mL^−1^ and temperature = 25 ± 0.2 °C. The lines in (a) and (d) are guides to the eye.

The water decontamination analysis was focused on Ni@ZnO@ZnS‐*Spirulina* because its optoelectronic characteristics and ROS generation are more adequate for solar photocatalysis than those of Ni@ZnO‐*Spirulina*, as was recently demonstrated for different ZnO and ZnO@ZnS morphologies.[qv: 4b,9b] However, note that, for comparison, the main photocatalytic properties of the Ni@ZnO‐*Spirulina* are given in **Figure** 3 and Figures S8–S10 in the Supporting Information.

The degradation of MB—10 ppm of MB in an algae culture medium—was used as a model to investigate the photomineralization activity of the Ni@ZnO@ZnS‐*Spirulina* under both artificial (light intensity of 680 ± 10 lx) and natural (average light intensity 1500 ± 300 lx) UV‐filtered (>400 nm) sunlight conditions. Both illumination conditions induce negligible photolysis (i.e., light induced degradation without catalyst; Figure S11, Supporting Information).

As can be seen in Figure 3a, the MB adsorption on the surface of Ni@ZnO@ZnS‐*Spirulina* after 90 min under dark conditions is 4.5%, indicating the good affinity of MB to ZnS. In general, both the large surface area and the adsorption affinity promote an increase in the density of active photocatalytic sites, thereby leading to enhanced photocatalytic activity.

Under UV‐filtered sunlight irradiation, the absorption intensity of MB decreased continuously with irradiation time, indicating the photooxidation of the MB (Figure 3a; Figure S8, Supporting Information). A colorless solution is achieved after 210 min of irradiation. The Ni@ZnO@ZnS‐*Spirulina* shows very high photocatalytic activity, with 88.9% and 86.5% of MB molecules degraded after 180 min under artificial and natural sunlight irradiation, respectively (**Table** 2).

**Table 2:** Photocatalytic performances of the Ni@ZnO@ZnS‐*Spirulina* under artificial and natural UV‐filtered sunlight irradiation (λ > 400 nm). Photocatalyst dosage = 0.5 mg mL^−1^ and temperature = 25 ± 0.2 °C

*Spirulina*	Artificial (light intensity of 680 ± 10 lx)			Natural (average light intensity 1500 ± 300 lx)		
	Irradiation time = 180 min		Irradiation time = 210 min	Irradiation time = 180 min		Irradiation time = 210 min
	MB degradation efficiency [%]	*k*_nor_ [min^−1^ g^−1^]	Mineralization efficiency [%]	MB degradation efficiency [%]	*k*_nor_ [min^−1^ g^−1^]	Mineralization efficiency [%]
Ni@ZnO@ZnS	88.9 ± 1.4	8.0	>99	86.5 ± 1.3	7.4	96.9 ± 1.9

It is well known that according to the Langmuir–Hinshelwood model, the photocatalytic degradation of MB at low concentrations follows pseudo first‐order kinetics with respect to MB concentration. Therefore, the plot of the logarithm of absorbance [− ln(*A_t_*/*A*_0_)] as a function of reaction time (*t*) (Figure S10, Supporting Information) allows the calculation of the apparent kinetic constant and consequently, the mass‐normalized kinetic constant (*k*_nor_), which can be used to compare the photocatalytic activity of the Ni@ZnO@ZnS‐Spirulina photocatalysts to the state‐of‐the‐art ZnO‐based photocatalysts.

The *k*_nor_ values of Ni@ZnO@ZnS‐*Spirulina* (8.0 and 7.4 min^−1^ g^−1^ under artificial and natural UV‐filtered sunlight, respectively) are comparable to or better than those of benchmark materials reported in the literature (Table S2, Supporting Information).[qv: 4b,9b,11] However, the photodegradation of persistent organic pollutants can lead to the formation of substances that are equally or more toxic. Consequently, determining the mineralization efficiency (complete degradation of organic pollutants to gaseous CO_2_ and inorganic ions) is an essential requirement before establishing Ni@ZnO@ZnS‐*Spirulina* as an efficient and competitive photocatalyst for water decontamination by solar photocatalysis. In the case of Ni@ZnO@ZnS‐*Spirulina* under artificial UV‐filtered sunlight irradiation, the total organic carbon (TOC) of the solution decreases from 10 to <0.05 ppm (detection limit of the TOC analyzer) after 210 min of irradiation, indicating that most of the organic molecules are mineralized (Figure 3b).

It is well known that hydroxyl radicals are the main actors in the photomineralization of POPs due to their high oxidation power. Nonetheless, the effects of hydroxyl radicals, superoxide ions, and photogenerated holes were visualized through trapping experiments performed during the photocatalytic degradation of MB. Singlet oxygen was not considered, as its formation in these conditions is negligible under these experimental conditions. Under UV‐filtered simulated sunlight, the degradation efficiency of the Ni@ZnO@ZnS‐*Spirulina* (88.9 ± 1.4%) decreased to ≈6%, ≈82%, and ≈48% in the presence of scavengers for hydroxyl radicals, superoxide ions, and photogenerated holes, respectively (Figure S12, Supporting Information). Therefore, as expected, hydroxyl radicals and photogenerated holes govern the mechanism of MB photodegradation under UV‐filtered simulated sunlight when Ni@ZnO@ZnS‐*Spirulina* is used as a photocatalyst.

To investigate the reusability of the Ni@ZnO@ZnS‐*Spirulina*, the photocatalysts were collected, washed, and dried after each photocatalytic reaction cycle (180 min) and reused for 25 consecutive cycles under artificial UV‐filtered sunlight irradiation. As can be seen in Figure 3c, the MB photodegradation efficiency remains virtually constant during the first seven successive cycles, which denotes an excellent stability of *Spirulina* photocatalysts. However, after this, the efficiency decreases slowly after each cycle, possibly as a consequence of weak photocorrosion (see below). After 25 cycles, the MB photodegradation efficiency is only 10% lower than that at the first cycle. After 25 reusability cycles, the BET surface area of the Ni@ZnO@ZnS‐*Spirulina* photocatalysts remained virtually constant (77.4 m^2^ g^−1^). In addition, FE‐SEM morphological analyses (Figure S13, Supporting Information) showed that the photocatalyst surface and the microalgal skeleton were not affected during these 25 reusability cycles. Therefore, Ni@ZnO@ZnS‐*Spirulina* exhibited a relatively high architectural stability without photocatalyst fragmentation. The reduction in the MB photodegradation (10%) efficiency over the course of 25 reusability cycles can mainly be ascribed to a loss of photocatalyst during its magnetic collection and cleaning after each cycle (or, less probably, to poisoning of the photocatalyst by the formation of nonmineralized carbonaceous species during MB photodegradation).

Additionally, the Zn(II) release was determined after 168 h (1 week) of artificial UV‐filtered sunlight irradiation to evaluate the photocorrosion activity and durability of the *Spirulina*‐based photocatalysts. As shown in Figure 3d, the Zn(II) release from the Ni@ZnO@ZnS‐Spirulina, and therefore the introduction of a new inorganic pollutant to the aqueous solution, is rather low. The dissolution is less than 5% of the initial ZnO@ZnS after 48 h under irradiation, thus exhibiting high anti‐photocorrosion properties promoted by the ZnS shell. Therefore, owing to its excellent photocatalytic efficacy, photocorrosion resistance, reusability, and recyclability the Ni@ZnO@ZnS‐*Spirulina* is an excellent and ecofriendly photocatalyst for water remediation with minimal adverse ecotoxicological effects. In contrast, the Ni@ZnO‐*Spirulina* showed much lower photocatalytic water decontamination efficiency and much faster degradation due to the photocorrosion effects (Figure 3; Figures S8 and S9, Supporting Information), thus highlighting the importance of the ZnS shell.

### 2.4 Bioethanol Production

Once the lifetime of the photocatalysts is reached, the structures can be recycled to produce biofuel. The inorganic shells of the hybrid Ni@ZnO@ZnS‐*Spirulina* photocatalyst can be easily removed and the biomass of the *Spirulina* can be efficiently converted to bioethanol in a simple and inexpensive simultaneous saccharification and fermentation process.[qv: 6e,12] Importantly, to show the innocuousness of the inorganic coating process, the ethanol production by the recycled and fresh *Spirulina* was compared.

The Ni@ZnO@ZnS‐*Spirulina* photocatalysts were collected after their application for water decontamination and dried. The remaining Ni, ZnO, and ZnS were dissolved using an acidic solution. Note that Ni(II) and Zn(II) can be easily recovered and separated by factorial crystallization and consequently, reutilized for the synthesis of new *Spirulina* photocatalysts.^13^ Subsequently, the microalgae were lysed by using a laboratory homogenizer to extract the glucose and glycogen using 8 g of dried *Spirulina* in 200 mL of yeast extract peptone medium (Formedium). The mixture was filtered, and the supernatant, which contained ≈26.9 g L^−1^ of glycogen and ≈0.7 g L^−1^ of glucose, was used to produce bioethanol.

The bioethanol production involved a novel simultaneous saccharification/fermentation process, where α‐glucosidase and α‐amylase were combined with a commercial yeast. First, the optimal concentration of enzymes and yeast were determined by using solutions containing 26 g L^−1^ of glycogen or 14 g L^−1^ of glucose to efficiently transform glycogen to glucose (saccharification) and glucose to ethanol (microbial fermentation). The best saccharification and fermentation results were obtained using 1.5 U L^−1^ α‐glucosidase, 3.5 U L^−1^ α‐amylase, and an initial yeast concentration of 1.55 × 10^7^ yeast mL^−1^.

**Figure 4:**
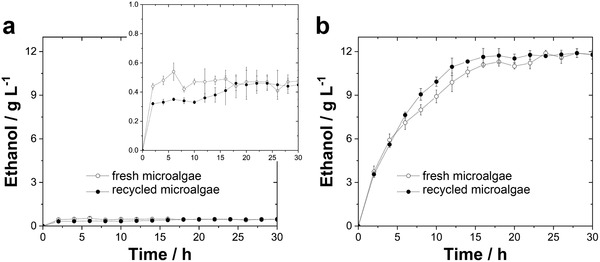
Ethanol production from the glycogen and glucose from fresh *Spirulina* (open circles) and *Spirulina* recycled from the Ni@ZnO@ZnS‐*Spirulina* photocatalysts used for water remediation (closed circles) in a) the absence of enzymes and b) the presence of 1.5 U L^−1^ α‐glucosidase and 3.5 U L^−1^ α‐amylase. The yeast concentration in each case is ≈1.55 × 10^7^ yeast mL^−1^ Ethanol Red. The data points and error bars are the mean values from three separate cultures and their corresponding standard deviation, respectively. The lines are guides to the eye.

To show the importance of performing the saccharification and fermentation simultaneously, the bioethanol production from recycled and fresh *Spirulina* was conducted in the presence or absence of enzymes. The direct fermentation without enzymes using ≈1.55 × 10^7^ yeast mL^−1^ of Ethanol Red lead to only 0.48 ± 0.07 and 0.45 ± 0.10 g L^−1^ of ethanol using fresh and recycled *Spirulina*, respectively, after 30 h of fermentation (**Figure** 4a). In contrast, simultaneous saccharification and fermentation (Figure 4b) offered an excellent way to obtain bioethanol. Namely, 11.8 ± 0.3 and 11.9 ± 0.3 g L^−1^ of ethanol were obtained using fresh and recycled *Spirulina*, respectively, after the same time period. The drastic enhancement of the production yield is due to the efficient conversion of glycogen to glucose in the presence of enzymes. The conversion efficiencies were 94.6 ± 2.5% and 94.4 ± 1.5% after 30 h of reaction at 31 ± 0.3 °C in the presence of α‐glucosidase and α‐amylase, respectively, resulting in glucose concentrations of 25.7 ± 0.7 and 25.7 ± 0.4 g L^−1^ using fresh and recycled *Spirulina* (Figure S14, Supporting Information), respectively.

The high bioethanol yield of the simultaneous saccharification/fermentation process is a clear evidence that the combination of enzymes and yeast is essential in obtaining bioethanol from the glycogen‐rich microalgae by microbial fermentation. Interestingly, the ethanol yield—based on the complete consumption of the microalgae glycogen and glucose—was ≈89% of the theoretical yield. The ethanol yield obtained in this work (≈0.4 L kg^−1^ of dried biomass) is comparable to the highest levels achieved using carbohydrate feedstocks (Table S3, Supporting Information).^14^ It is important to emphasize that the yield of recycled *Spirulina* was virtually identical to that of fresh *Spirulina*, indicating that metallization and water decontamination processes did not damage the glycogen and glucose of the microalgae.

### 2.5 Closing the Cycle—CO_2_ Fixation

ZnO‐based hybrid photocatalysts based on microalgae can be used for the efficient mineralization of persistent organic pollutants and then recycled to directly produce bioethanol by a simultaneous saccharification and fermentation process, releasing only CO_2_ gas as residue. By using this gas to close the circle, i.e., eliminating the produced residues, a green circular strategy can be offered for water decontamination and bioethanol production. Consequently, as the last stage to close the circular process and start a new cycle, new microalgae are cultivated by using the i) clean water and CO_2_ gas obtained from photocatalytic water decontamination and ii) CO_2_ gas generated during saccharification and fermentation (Scheme 1). The photosynthesis simultaneously fixes carbon into microalgae and yields vital products such as oxygen and glycogen in a single solar‐driven process, converting Ni@ZnO/ZnS‐Spirulina in a zero‐carbon‐emission route for sunlight photocatalytic water decontamination and bioethanol production.

## 3 Conclusions

Summarizing, a holistic circular process, mimicking the life cycle, using a hybrid Ni@ZnO@ZnS‐*Spirulina plantensis* var lonar microalgae, which integrates CO_2_ fixation, water remediation, and bioethanol production with minimal ecotoxicological effect, has been successfully demonstrated.

The Ni@ZnO@ZnS‐Spirulina microalgae exhibited an excellent photocatalytic performance in the mineralization of persistent organic pollutants under sunlight irradiation, with excellent durability. The solar photocatalytic efficacy was comparable to or better than those of benchmark materials reported in the literature. Importantly, the clean water and the carbon dioxide produced during mineralization could be exploited for the cultivation of new microalgae within the circular process.

The recycling of Ni@ZnO@ZnS‐Spirulina provided metal ions that can be recovered and reused to synthesize new photocatalysts, while the microalgae skeleton was later exploited for bioethanol production, thereby minimizing the residues of the circular process. We showed that simultaneous saccharification and fermentation of the biomass allows for the direct production of bioethanol without pretreatments, presenting yields that are comparable to the highest levels achieved using carbohydrate feedstocks.

Thus, the magnetic and photocatalytic plated microalgae hybrids offer a green circular strategy to integrate CO_2_ fixation, water remediation, and bioethanol production, which can be easily scaled for industrial applications.

## Conflict of Interest

The authors declare no conflict of interest.

## Supporting information

Supporting Information
