# Supplementary material for: Hybrid Ni@ZnO@ZnS‐Microalgae for Circular Economy: A Smart Route to the Efficient Integration of Solar Photocatalytic Water Decontamination and Bioethanol Production
Source: Adv Sci (Weinh). 2019 Dec 12;7(3):1902447. doi: 10.1002/advs.201902447 (PMC7001628; doi:10.1002/advs.201902447)
Supplement: Supplementary file 1 — Supporting Information [file ADVS-7-1902447-s001.pdf]

Copyright WILEY-VCH Verlag GmbH & Co. KGaA, 69469 Weinheim, Germany, 2019.

ADVANCED SCIENCE Open Access

Supporting Information

for *Adv. Sci.,* DOI: 10.1002/advs.201902447

Hybrid Ni@ZnO@ZnS-Microalgae for Circular Economy: A Smart Route to the Efficient Integration of Solar Photocatalytic Water Decontamination and Bioethanol Production

Albert Serrà, Raül Artal, Jaume García-Amorós, Borja Sepúlveda, Elvira Gómez, Josep Nogués, and Laetitia Philippe*

Copyright WILEY-VCH Verlag GmbH & Co. KGaA, 69469 Weinheim, Germany, 2019.

Supporting Information

Hybrid Ni@ZnO@ZnS-Microalgae for circular economy: A smart route to the efficient integration of solar photocatalytic water decontamination and bioethanol production

*Albert Serrà, * Raul Artal, Jaume García-Amorós, Borja Sepúlveda, Elvira Gómez, Josep Nogués, and Laetitia Philippe*

1. Experimental Section

Microalgae, cultivation conditions, and biochemical analysis. The cyanobacteria *Spirulina platensis var lonar* were cultured in an algal culture medium – 4.5 g L^{-1} of $NaHCO_3$ (Fluka-Chemika, > 99%), 0.5 g L^{-1} of K_2HPO_4 (Fluka, > 99%),1.0 g L^{-1} of K_2SO_4 (Sigma-Aldrich, >99 %), 1.0 g L^{-1} of NaCl (Sigma-Aldrich, > 99.5%), 1.2 g L^{-1} of $MgSO_4$ (Sigma-Aldrich, > 99.5%), 2.5 g L^{-1} $NaNO_3$ (Sigma-Aldrich, > 99.0%), 0.04 g L^{-1} $CaCl_2$ (Sigma-Aldrich, > 97%), 0.01 g L^{-1} of $FeSO_4 \cdot 7H_2O$ (Sigma-Aldrich, > 99%) – with intermittent mechanical agitation (80 rpm; 30 min-15 min on-off cycles) under natural sunlight (average light intensity of 1350 ± 450 lx) at 30°C. The intermittent mechanical stirring (80 rpm) was maintained during the night with 15 min-180 min on-off cycles. The pH was controlled, readjusted and maintained at a value of 9.8. The microalgae were also cultivated in the nitrate-deficient – 4 mM $NaNO_3$ (Sigma-Aldrich, > 99%) – algal culture medium to improve their glycogen content during 5 days prior to being used as a biotemplate.

The glucose and glycogen content of the cyanobacteria *Spirulina platensis var lonar* were extracted and determined as follows: (i) 1 g of dried microalgae was washed twice with 2M H_2SO_4 (Sigma-Aldrich, 95%), cold phosphate-buffered saline (PBS, 0.01 M phosphate buffer, 0.0027 M KCl and 0.137 M NaCl, pH 7.4) and dispersed in 40 mL of PBS containing 1X

SigmaFast protease inhibitor tablets (#S8820; Sigma-Aldrich); (ii) microalgae were then physically lysed in order to extract glucose and glycogen by using a laboratory homogenizer (20,000 rpm, 3 min). To prevent excessive heating, the mechanical disruptions were applied in multiple short on-off cycles (30 s) to a sample immersed in an ice bath; (iii) homogenates were centrifuged at 13,000 rpm at 4°C for 10 min, and supernatants were recovered for glucose and glycogen analysis and filtered through 0.2 μm syringe filters. Next, the glucose and glycogen content were determined using a glucose (HK) assay kit (#GAGO20, Sigma-Aldrich) and glycogen assay kit (#MAK016, Sigma-Aldrich), respectively.

Synthesis of microalgae-based photocatalyst. The biotemplating process combines (**Scheme S1):**

- **Microalgae fixation.** The microalgae were collected by a nylon mesh filter (100 μm mesh) and washed twice with PBS. Then, the filtered microalgae were resuspended in 50 mL of 10% glutaraldehyde (#G5882, Sigma-Aldrich) in PBS for 6 h at 25°C to fixate the microalgae and prevent fragmentation. After the fixation treatment, the microalgae were filtered and washed first with PBS and then with deionized water. The fixed microalgae can be stored in cool, dark conditions and be used after several months.
- **Pd catalyzation:** To catalyze the metallization of microalgae by means of nickel electroless deposition, the fixed microalgae were activated using a palladium catalyzation bath. The palladium bath was prepared as follows: (i) 50 mL of a 5 mM $PdCl_2$ (Sigma-Aldrich, 99%) + 0.2 M HCl (Sigma-Aldrich, 37%) solution was prepared at 25°C; (ii) 0.2 g of $SnCl_2$ (Sigma-Aldrich, 98%) were dissolved and stirred for 10 min; (iii) 1.6 g of $SnCl_2$ (Sigma-Aldrich, 98%) were added and dissolved. Then, the catalytic microalgae were prepared by resuspending 1 g of fixed, dried microalgae in 50 mL of palladium catalyzation bath for 25 min at 35°C. Next, the catalyzed microalgae were filtered and washed with 1M HCl (x3), PBS (x2) and deionized water. Then, the activated

microorganisms can be stored in cool, dark conditions + N_2 atmosphere and used after several months.

- **Nickel electroless deposition.** The composition of the nickel electroless bath was 0.2 M sodium citrate tribasic dehydrate (Sigma-Aldrich, > 99%) + 0.1 M $NiSO_4{\cdot}7H_2O$ (Sigma-Aldrich, > 99%) + 0.05 M borane dimethylamine complex (Sigma-Aldrich, 97%). The electroless bath was prepared as follows: (i) 50 mL of a 0.2 M sodium citrate tribasic dehydrate (Sigma-Aldrich, > 99%) + 0.1 M $NiSO_4{\cdot}7H_2O$ (Sigma-Aldrich, > 99%) solution were prepared; (ii) then, this aqueous solution was stirred (400 rpm) for 30 min; and prior to starting the electroless deposition process, (iii) borane dimethylamine complex was added; then (iv) the pH was adjusted with 0.5 M NaOH (Sigma-Aldrich, > 98%). Prior to beginning the electroless deposition, the electroless bath was heated and maintained at 70°C. Next, 1 g of the catalyzed microalgae was dispersed in 50 mL of the electroless bath and stirred with N_2 bubbling for 10 min at 70°C to form a nickel shell on the microalgae, resulting in the Ni-*Spirulina* core@shell structure. Next, nickel microalgae were filtered and washed with deionized water (x5).
- **Zinc oxide chemical deposition.** The nickel metallized microalgae were recovered with a ZnO thin layer by means of a chemical deposition process. 1 g of Ni-*Spirulina* core@shell was immersed and stirred with N_2 bubbling in an aqueous solution containing 0.01 M $Zn(NO_3)_2$ + 0.01 M borane dimethylamine complex for 4 min at 70°C to form a ZnO thin layer on Ni-*Spirulina*, resulting in an onion-like Ni@ZnO-*Spirulina* microstructure. Then, the microstructures were filtered and washed with deionized water (x5).
- **Zinc sulfide growth (sulfidation).** After the deposition of ZnO, the microstructures were immersed in an aqueous solution of 30 mM thioacetamide (Sigma-Aldrich, 98%) for 4 h at 85°C to form a ZnS shell, resulting in an onion-like Ni@ZnO@ZnS-*Spirulina* microstructure. Lastly, the microstructures were filtered and washed with deionized water (x5).

Characterization of the microalgae-based photocatalyst. Field-emission scanning electron microscopes (FE-SEM, Hitachi S-4800 and H-4100FE) equipped with an energy-dispersive X-ray spectroscopy detector were used to characterize the morphology, elemental composition, and architecture. The specific surface areas, based on the Brunauer–Emmett–Teller (BET) method from N_2 adsorption-desorption isotherms at 77 K, were measured using a Micrometrics Tristar-II. X-ray diffraction (XRD, Bruker D8 Discovery diffractometer) in the Bragg–Brentano configuration with Cu K_α radiation was used to determine the crystal phases. The X-ray photoelectron spectroscopy (XPS) measurements were carried out using a PHI ESCA-5500 Multi-technique system (Physical Electronics) in a base pressure of 5 10^{-10} mbar using a monochromatic X-ray source (Al K_α a line of 1486.6 eV and 350 W), placed perpendicular to the analyzer axis and calibrated using the $Ag3d_{5/2}$ line with a full width at half maximum of 0.8 eV. The optical and electronic properties and the light absorption ability were characterized by UV-vis spectroscopy (PerkinElmer Lambda 900 UV spectrophotometer), photoluminescence spectroscopy – custom made set up, composed of an LED source with emission at 365 nm (M365FP1, Thorlabs), which was band-pass filtered (FB360-10, Thorlabs) for excitation – and transient photocurrent densities. The backscattered PL was long-pass filtered (FEL0400, Thorlabs) and it was detected with an Andor 193i spectrometer equipped with an Andor Idus camera. The photocurrent tests were performed with a PGSTAT30 potentiostat-galvanostat Autolab and the NOVA software (Version 2.1.4; Metrohm Autolab) using a three-electrode system with a Pt wire as the counter electrode, an Ag/AgCl/KCl (3 M)/ Na_2SO_4 (0.5 M) electrode as the reference, and *Spirulina*-based photocatalysts (300 µL suspension; photocatalyst dosage = 0.5 mg mL^{-1}) deposited and dried on a 0.5 cm × 0.5 cm indium–tin–oxide conducting glass as the working electrode. The electrolyte used for all the experiments was 0.5 M Na_2SO_4 (pH 7). The photocurrent response of each photocatalyst was recorded at 0.1 V versus the Ag/AgCl/KCl (3 M)/ Na_2SO_4 (0.5 M)

electrode under artificial UV-filtered sunlight in (30 s – 20 s) on-off cycles. Controlled irradiation was provided by a 75 W Xe lamp (light intensity of 680 ± 10 lx).

Water decontamination. Photocatalytic mineralization of persistent organic pollutants

- **Identification of reactive oxygen species (ROS):** Chemical selective radical quenchers were used to determine the formation of hydroxyl radicals ($^{\bullet}OH$), oxygen superoxide ions (O_2^-), and singlet oxygen ($^1O_2$) by the ZnO-based photocatalysts under UV-filtered simulated sunlight (> 400 nm, light intensity of 680 ± 10 lx) at 25°C. The formation of hydroxyl radicals was determined by spectroscopically monitoring the consumption of 8 µM of fluorescein sodium salt (Supelco/Sigma-Aldrich), assuming zero order kinetics and equimolar reaction stoichiometry between hydroxyl radicals and fluorescein molecules. Specifically, the fluorescein concentration was determined by following the time-dependent reduction of the photoluminescence peak at 515 nm (λ_{ex} = 303 nm), measured in quartz cuvettes using an AMINCO-Bowman Series 2 spectrofluorometer. The formation of oxygen superoxide ions was spectroscopically determined by following the reaction of superoxide ions with 100 µM 2,3-Bis(2-methoxy-4-nitro-5-sulfophenyl)-2*H*-tetrazolium-5-carboxanilide (XTT) (Supelco/Sigma-Aldrich, > 90%); specifically, the 475 nm absorption peak characteristic of XTT-formazan, the product of XTT reduction by superoxide ions, was monitored using a UV-1800 Shimadzu UV-vis spectrophotometer. Lastly, the formation of singlet oxygen ($^1O_2$) was evaluated using the highly-selective singlet oxygen sensor green (SOSG) reagent (5 mM in methanol; Invitrogen) and monitoring the time-dependent photoluminescence intensity at 535 nm (λ_{ex} = 488 nm) using an AMINCO-Bowman Series 2 spectrofluorometer. The intensity of this peak corresponds to the formation of the endoperoxide SOSG-$^1O_2$, which is produced by the reaction of the anthracene moiety part of the SOSG reagent with singlet oxygen.

- **Photocatalytic mineralization of persistent organic pollutants (POPs):** The photocatalytic activities of the Ni@ZnO- and Ni@ZnO@ZnS-*Spirulina* were evaluated by following the photocatalytic degradation and mineralization of a single pollutant solution of 10 ppm of methylene blue (Sigma-Aldrich, > 97 %) under artificial and natural UV-filtered sunlight irradiation. Pollutant solutions were prepared in the algae culture medium. Controlled irradiation of reactor was provided by using a 75 W Xe lamp setup (light intensity of 680 ± 10 lx) or natural sunlight (average light intensity 1500 ± 300 lx) irradiation for artificial and natural UV-filtered irradiation, respectively. A 400 nm UV cut-off filter was used for each experiment to remove the UV irradiation ($\lambda < 400$ nm). Pollutant solutions were maintained under controlled conditions during the photocatalyst process (photocatalyst dosage = 0.5 mg mL^{-1} and temperature = 25 ± 0.2°C). Prior to the photocatalytic reaction, the organic pollutant solutions containing photocatalysts at 25°C were kept in the dark-room for 90 min to establish an adsorption-desorption equilibrium. The pollutant solutions were then exposed to UV-filtered irradiation under air bubbling and the photoremediation process was followed using the following methods: (i) the temporal evolution of the reduction in the maximum absorption peak intensity of methylene blue (662 nm) under irradiation during 120 min using a UV-vis spectrophotometer (UV-1800, Shimadzu Corporation) in a quartz cuvette with an optical length of 1 cm; and (ii) the mineralization efficiency, by comparing the initial total organic carbon (TOC) content with the temporal evolution of TOC content for 210 min, by using the high-temperature combustion method on a catalyst (Pt-Al_2O_3) in a tubular flow microreactor operated at 680°C, with a stream of hydrocarbon free air to oxidize the organic carbon, using a TOC-V_{CSH} equipment (Shimadzu) with a high-sensibility column. The effect of ROS and photogenerated holes on the photodegradation of MB was identified using selective radical scavengers. In these experiments, a 10 ppm MB

solution that had achieved adsorption-desorption equilibrium in dark conditions was prepared, and various radical scavengers were independently added to attain the following concentrations: (i) 1 mM isopropyl alcohol (Sigma-Aldrich, >99.7%) – a quencher of hydroxyl radicals; (ii) 1 mM benzoquinone (Sigma-Aldrich, >99.7%) – a quencher of superoxide ions; and (iii) 1 mM triethanolamine (Sigma-Aldrich, >99%) – a quencher of photogenerated holes. Next, the reactor was irradiated using UV-filtered simulated sunlight (> 400 nm, light intensity of 680 ± 10 lx) at 25 °C for 180 min and the maximum absorption peak intensity of MB at 662 nm was measured.

- **Reusability and photostability of the *Spirulina*-based photocatalysts:** The reusability of Ni@ZnO- and Ni@ZnO@ZnS-*Spirulina* was evaluated by measuring the photodegradation efficiency ($A_{180\,min}/A_{0\,min}$) of 10 ppm methylene solution for 25 consecutive cycles under artificial UV-filtered irradiation. The photocatalyst stability after 25 reusability cycles was evaluated in terms of BET surface area and morphological and architectural observations conducted using FE-SEM microscopy. The long-term stability of Ni@ZnO- and Ni@ZnO@ZnS-*Spirulina* was evaluated by immersing each photocatalyst in algae medium for 7 days under natural sunlight irradiation, and then conducting the photocatalytic degradation of methylene blue and measuring the Zn(II) concentration in the algae medium with the pre-described procedures. The Zn(II) concentration was examined by a spectrophotometric method using Zincon monosodium salt (Sigma-Aldrich) in borate buffer (50 mM, pH=9). The absorbance at 620 nm, associated to the Zn(II)-bound Zincon complex, was measured using a UV-vis spectrophotometer (UV-1800, Shimadzu Corporation).

*Bioethanol production***.** Bioethanol production from (i) the fresh *Spirulina platensis var lonar* and (ii) the recycled Ni@ZnO- and Ni@ZnO@ZnS-*Spirulina* denuded of the covering layer was studied. For that purpose, the above two samples were subjected to saccharification,

fermentation, or simultaneous saccharification and fermentation processes. The same steps were followed for the fresh and the recycled microalgae:

1. The ethanol production process started by removing the Ni@ZnO- and Ni@ZnO@ZnS-*Spirulina* covering and the glycogen extraction by following the procedure described in "*microalgae, cultivation conditions, and biochemical analysis*" section, yielded a blue solution containing the extracted glycogen and glucose.
2. 100 mL of microalgae glycogen and glucose were mixed with 100 mL of YEP fermentation media (Formedium™) – 10 g L^{-1} of yeast extract + 20 g L^{-1} of Peptone.
   - For saccharification: Enzymes were added to the saccharification media to obtain a 1.5 U L^{-1} α-Glucosidase from *Saccharomyces cerevisiae* (#G5003, Sigma-Aldrich) and 3.5 U L^{-1} α-Amylase from porcine pancreas (#A3176, Sigma-Aldrich) concentration.
   - For fermentation: An initial concentration of ∼1.55 10^7 yeast mL^{-1} of Ethanol Red^R – hybrid yeast – provided by Société Industrielle Lesaffre, Division Leaf are added to the fermentation media. Yeasts were prepared following this process: Yeasts were first rehydrated during 30 min in 5 times its weight of sterile water at 31°C. Prior to be used, the hydrated yeasts were first (i) grown in YPD medium (Formedium™) – 10 g L^{-1} of yeast extract + 20 g L^{-1} of Peptone + 20 g L^{-1} of Glucose – at 31°C under CO_2 bubbling for 30 h; and (ii) collected by centrifugation (10,000 rpm) for 3 min at 25°C, washed twice with distilled water, and then inoculated in the reaction medium. The yeast concentration was controlled and stablished by using a Neubauer Chamber.
   - For simultaneous saccharification and fermentation: Enzymes were added to the saccharification media to obtain a 1.5 U L^{-1} α-Glucosidase and 3.5 U L^{-1} α-Amylase

concentration and yeast were introduced to obtain an initial concentration of ∼1.55 10^7 yeast mL^{-1} of Ethanol Red^R.

The saccharification, fermentation, or simultaneous saccharification and fermentation processes were conducted under magnetic stirring (300 rpm) at 31 ± 0.3°C. The time-dependent evolution of glucose, glycogen, and ethanol content was determined during the 30 h period of each experiment by using glucose (HK) assay kit (#A3176, Sigma-Aldrich), glycogen assay kit (#MAK016, Sigma-Aldrich), and gas chromatography – GC-2010 chromatograph (Shimadzu) equipped with flame ionization detector and a HP-5 column (30 m x 0.32 mm of intern diameter, 0.25 µm of film thickness).

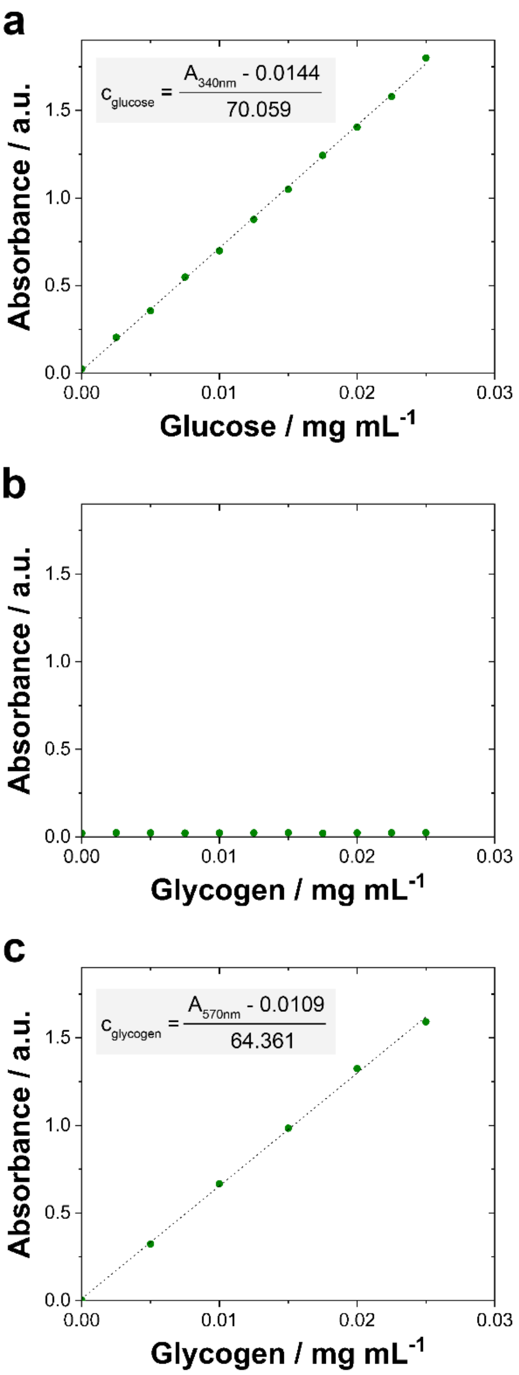

Figure S1: Calibration curves of the absorbance versus concentration for (a) glucose and (b, c) glycogen standards using (a, b) glucose (HK) assay kit (#GAGO20, Sigma-Aldrich) – absorbance measured at 340 nm – and (c) glycogen assay kit (#MAK016, Sigma-Aldrich) – absorbance measured at 570 nm.

2. Biochemical composition of the *Spirulina platensis*

Table S1: Biochemical composition (glycogen and glucose) of the fresh fixed *Spirulina* platensis and the *Spirulina* photocatalyst cultivated in different media. Composition based on dry-microalgae weight (wt. %).

Material	Glycogen [wt. %]		Glucose [wt. %]	
	30 mM $NaNO_3$	4 mM $NaNO_3$	30 mM $NaNO_3$	4 mM $NaNO_3$
**Fixed *Spirulina* platensis (as-prepared)**	28.5 ± 1.1	68.1 ± 1.3	1.8 ± 0.3	1.9 ± 0.3
***Spirulina* photocatalyst (as-prepared)**	27.2 ± 1.6	67.4 ± 2.1	2.1 ± 0.4	1.7 ± 0.2
***Spirulina* photocatalyst (after 2 weeks)**	26.1 ± 2.4	66.8 ± 2.2	2.0 ± 0.5	1.5 ± 0.4

3. *Spirulina*-based biotemplating process

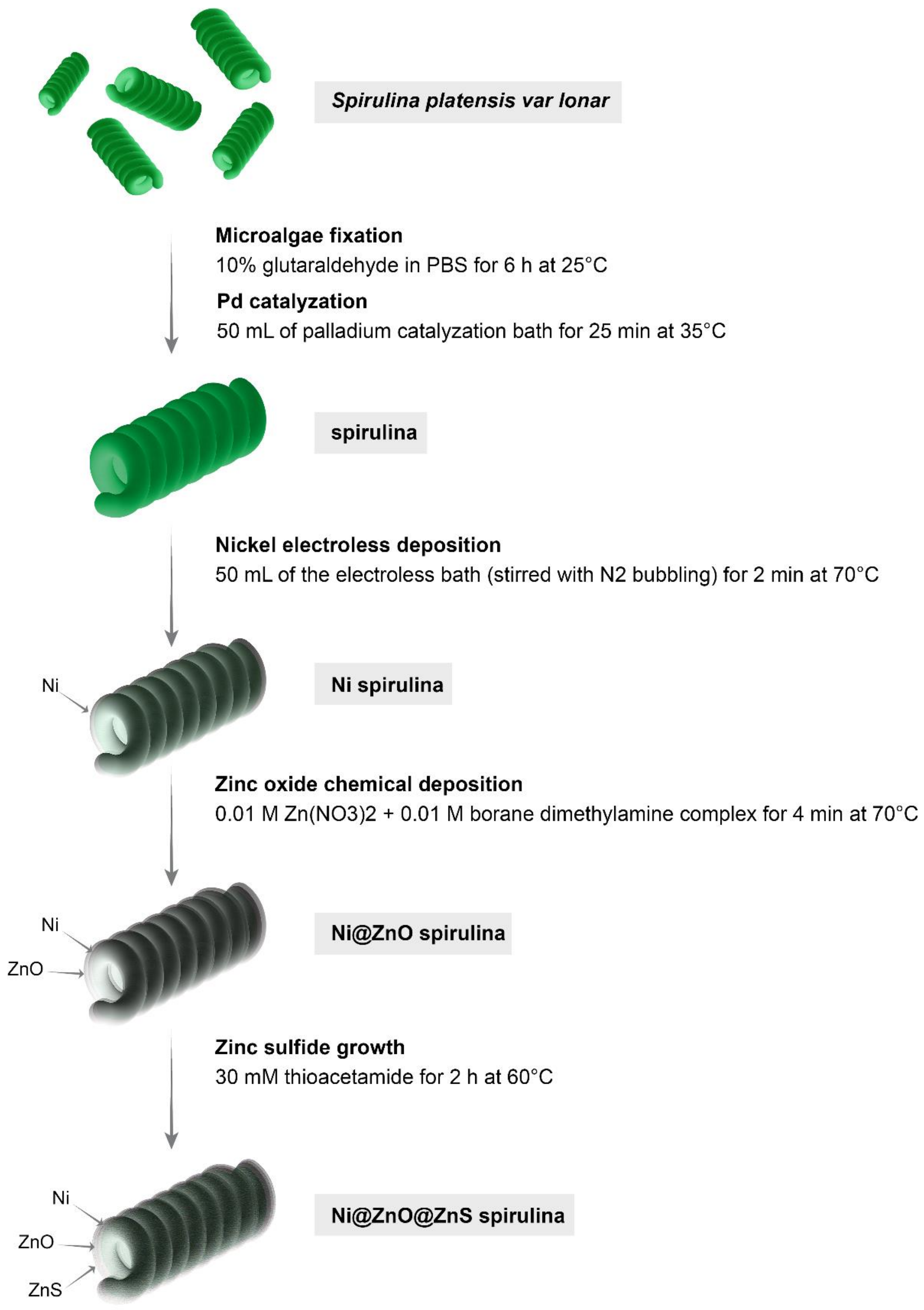

Scheme S1: Schematic illustration of the biotemplating process of Ni@ZnO and Ni@ZnO@ZnS *Spirulina*.

4. Photograph of magnetic collection of the hybrid microalgae

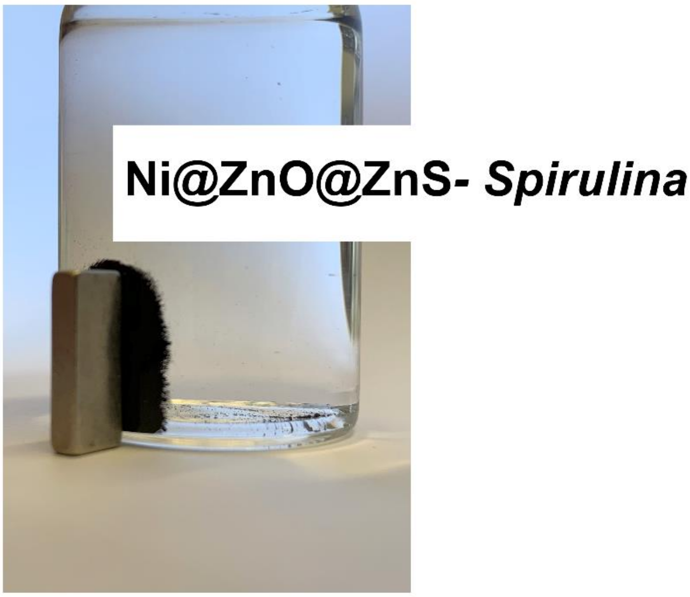

Figure S2: Photograph of magnetic collection of the hybrid microalgae.

5. Brunauer-Emmett-Teller (BET) surface areas

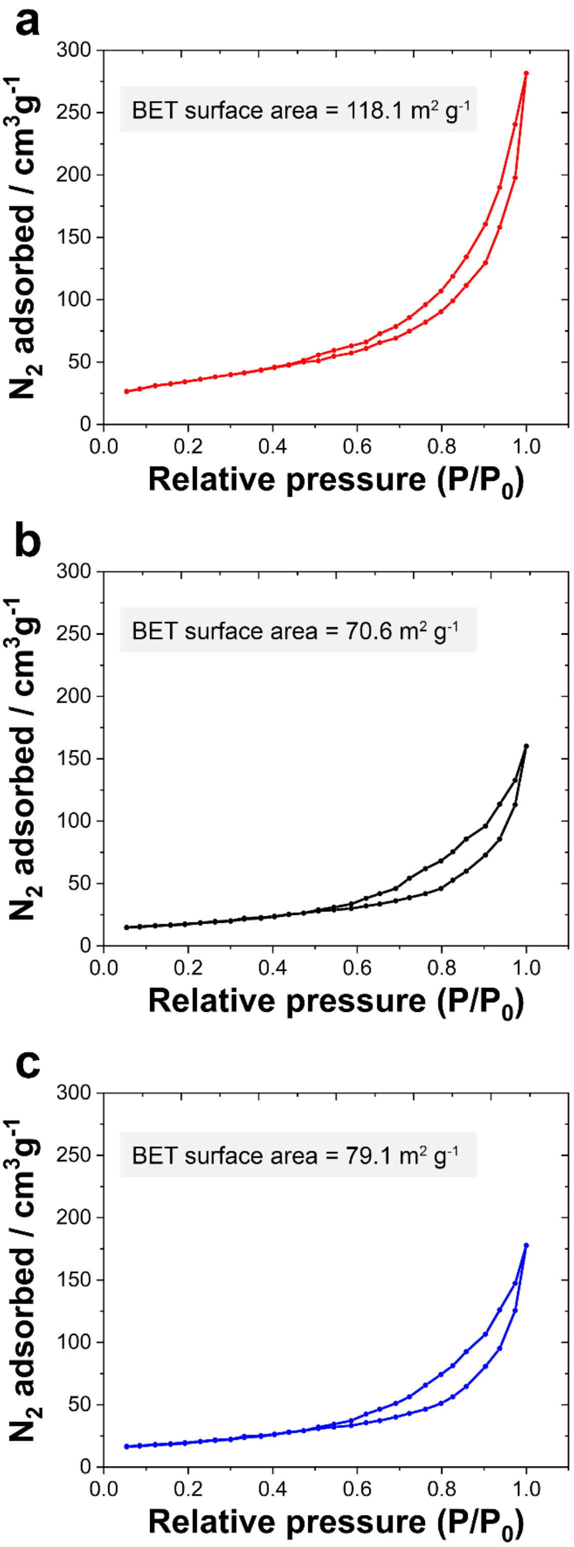

Figure S3: N_2 adsorption-desorption isotherms of the (a) Ni, (b) Ni@ZnO-, and (c) Ni@ZnO@ZnS-*Spirulina*.

6. X-Ray diffraction patterns of the *Spirulina*-based *a* photocatalysts

The XRD diffraction patterns are displayed in **Figure S4**. The Ni *Spirulina* XRD pattern exhibited a single broad peak (around 2θ 44.5°), suggesting the formation of a nanocrystalline Ni coating on the microalgae. On the other hand, the ZnO layer (Ni@ZnO-*Spirulina*) was polycrystalline, with clear diffraction peaks at $2\theta = 31.79°$(100), 34.42°(002), 36.25°(101), 56.60°(110), and 62.86°(103) (in agreement with wurtzite ZnO; JCPDS card 36-1451). The XRD pattern after sulfidation confirmed the formation of a thin ZnS layer on ZnO (Ni@ZnO@ZnS-*Spirulina*), with two extra diffraction peaks appearing at $2\theta = 28.55°$(111) and 33.87°(200), matching the cubic ZnS blende structure (JCPDS card 65-1691).

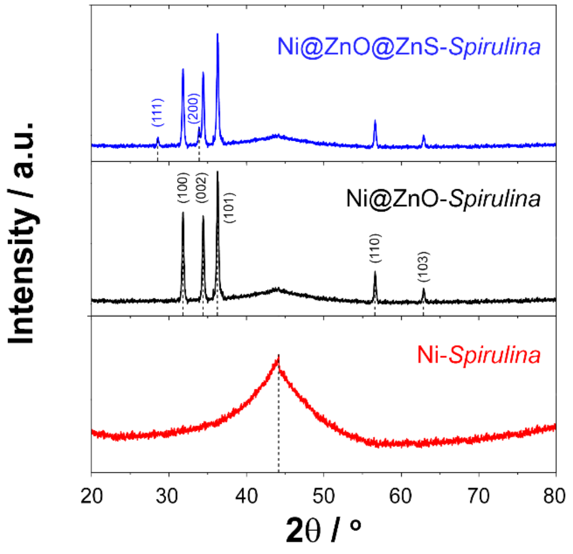

Figure S4: XRD patterns of Ni, Ni@ZnO, and Ni@ZnO@ZnS *Spirulina*.

7. X-ray photoelectron spectroscopy of *Spirulina* photocatalysts

The chemical states of Ni and Zn on the multi-layered core@shell microstructures were investigated in detail by analyzing the XPS spectra of Ni 2p, Zn 2p, O 1s, and S 2p (**Figure S5**). The XPS analysis indicated: (i) the formation of a Ni metallic, which was partially and superficially oxidized (probably a mixture of NiO and NiO_xH_y), for the Ni-*Spirulina*; (ii) the growth of ZnO (note that neither Ni nor NiO were detected for the Ni@ZnO- or Ni@ZnO@ZnS-*Spirulina* structures); (iii) the deposit of ZnO and ZnS for the Ni@ZnO- or Ni@ZnO@ZnS-*Spirulina* structures, due to the detection of Zn $2p_{3/2}$ peaks at 1021.6 and 1022.3 eV, respectively (these binding energies correspond to Zn(II) ions in an oxygen and sulfur environment, respectively); and (iv) the sulfidation of ZnO, since the O 1s peak was not detected on the Ni@ZnO@ZnS-*Spirulina* XPS spectra, while an asymmetric S 2p feature deconvoluted into two sub-peaks (corresponding to S $2p_{3/2}$ and S $2p_{1/2}$) appears.

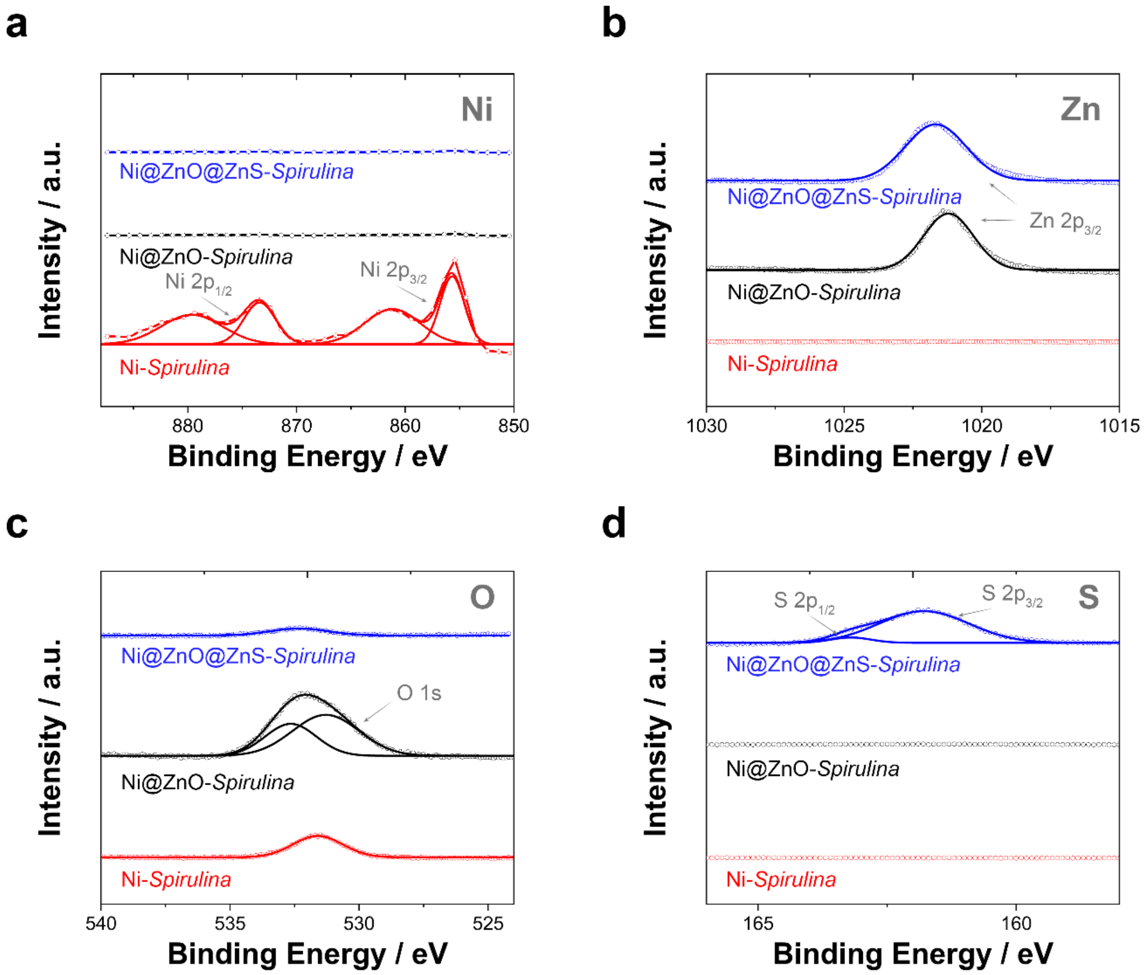

Figure S5: XPS spectra of (a) Ni 2p, (b) Zn 2p, (c) O 1s, and (d) S 2p of the Ni-, Ni@ZnO-, and Ni@ZnO@ZnS-*Spirulina*.

8. Tauc plots of Ni@ZnO and Ni@ZnO@ZnS photocatalysts

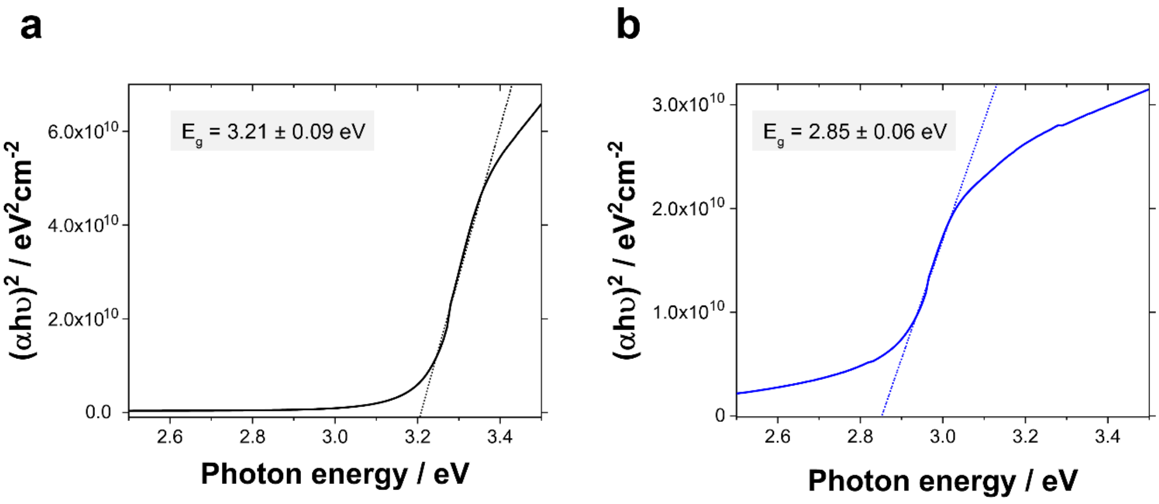

Figure S6: Tauc plots from the UV-vis analysis of the (a) Ni@ZnO- and (b) Ni@ZnO@ZnS-*Spirulina* photocatalysts.

9. Reactive oxygen species identification

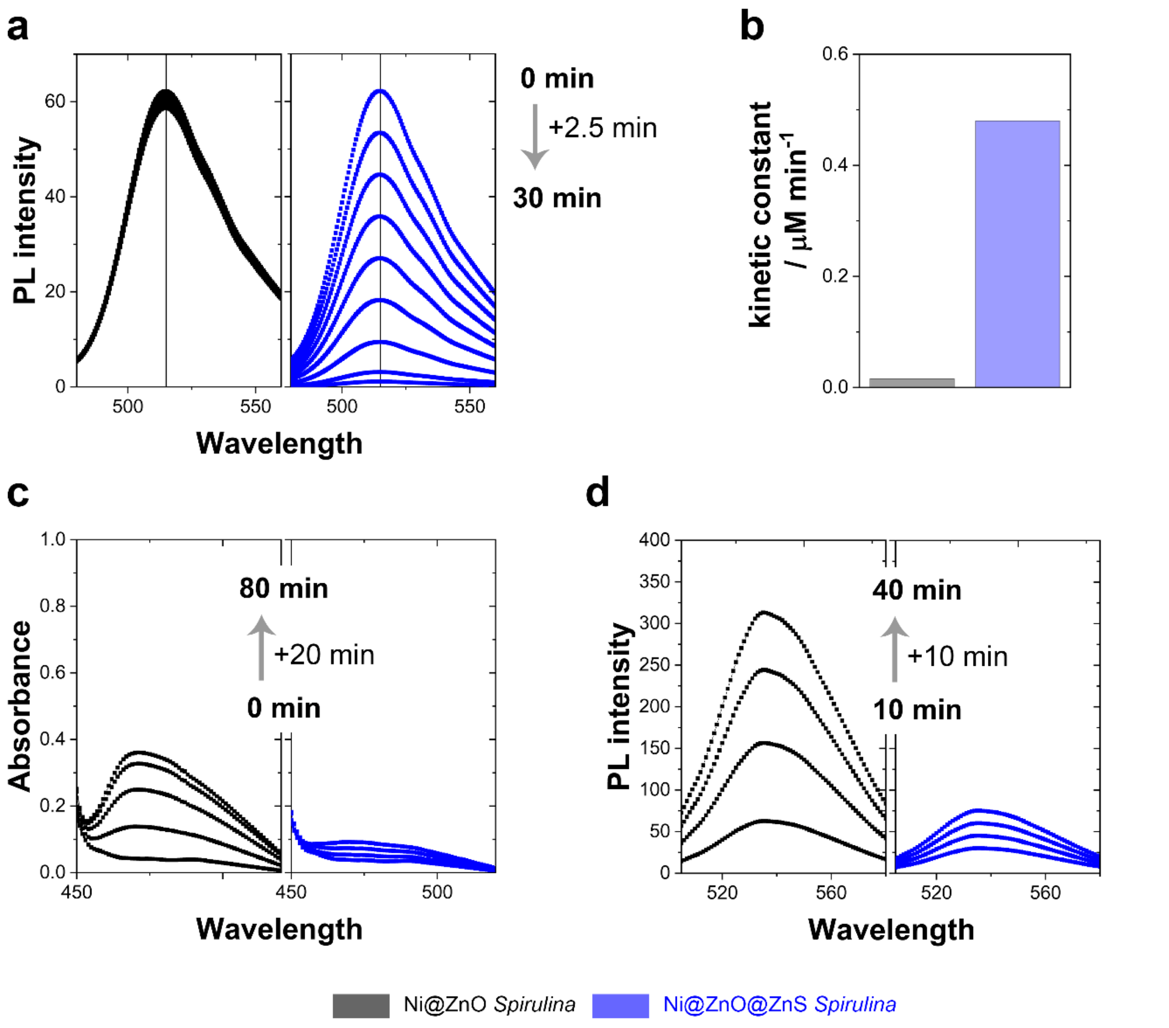

Figure S8: Time-dependent photoluminescence spectra of (a) 8 μM fluorescein, which indicates the consumption of generated hydroxyl radicals, and (d) endoperoxide formation, which indicates the formation of singlet oxygen, using ZnO-based hybrid photocatalysts. (b) Kinetic constant of hydroxyl radical formation using ZnO-based hybrid photocatalysts. (c) Time-dependent UV-vis spectra of the formation of XTT-formazan, which indicates the formation of oxygen superoxide ions using ZnO-based hybrid photocatalysts. Photocatalyst dosage = 0.5 mg mL^{-1}; temperature = 25 ± 0.2°C; Irradiation: UV-filtered simulated sunlight (> 400 nm, light intensity of 680 ± 10 lx).

10. Photocatalytic degradation of Methylene Blue

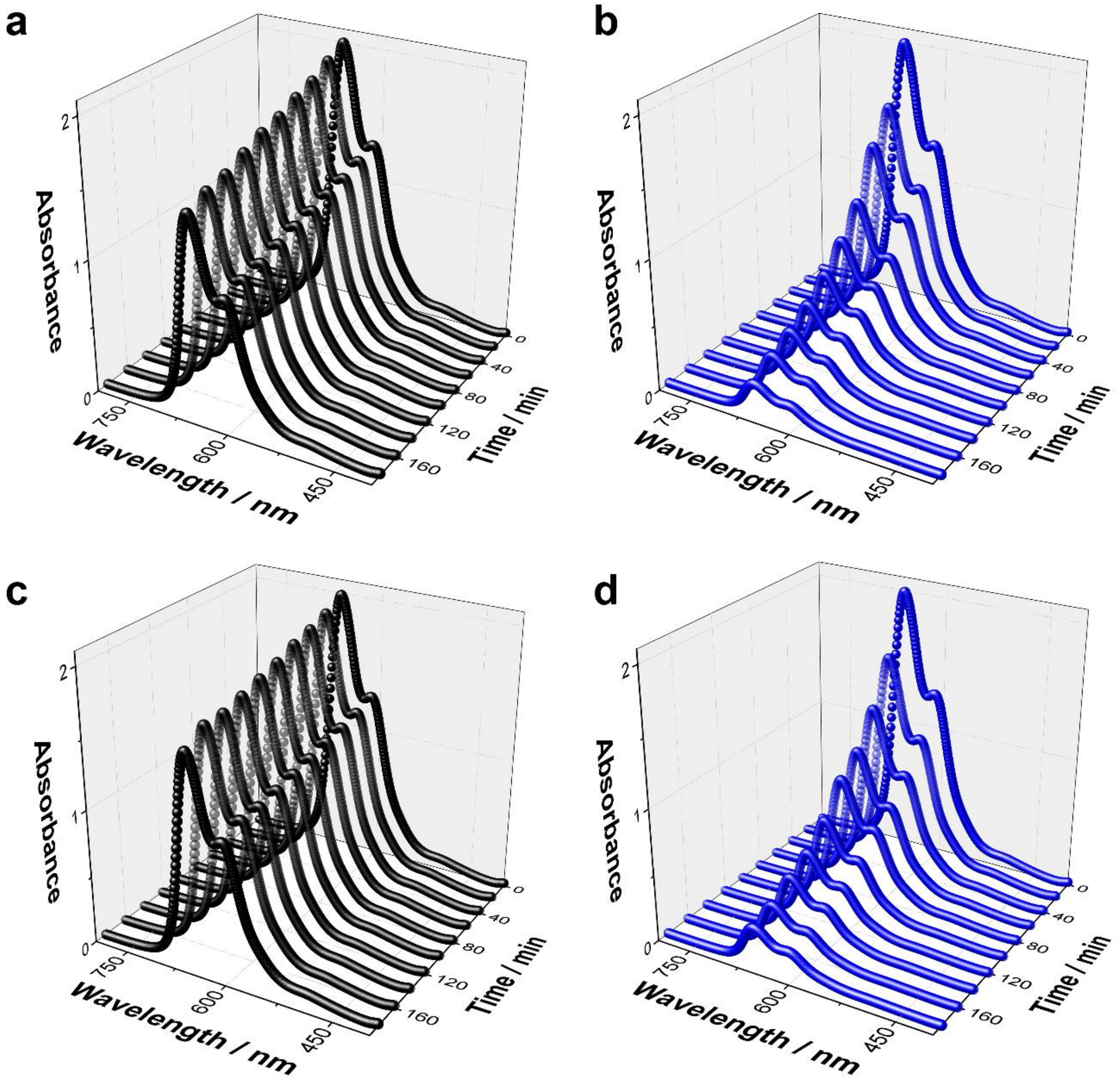

Figure S9: Time-dependent UV-vis spectra of the MB photocatalyzed degradation under UV-filtered (λ > 400 nm) (a, b) artificial and (c, d) natural (λ > 400 nm) sunlight using (a, c) Ni@ZnO- and (b, d) Ni@ZnO@ZnS-S*pirulina* (photocatalyst dosage: 0.5 mg mL^{-1}).

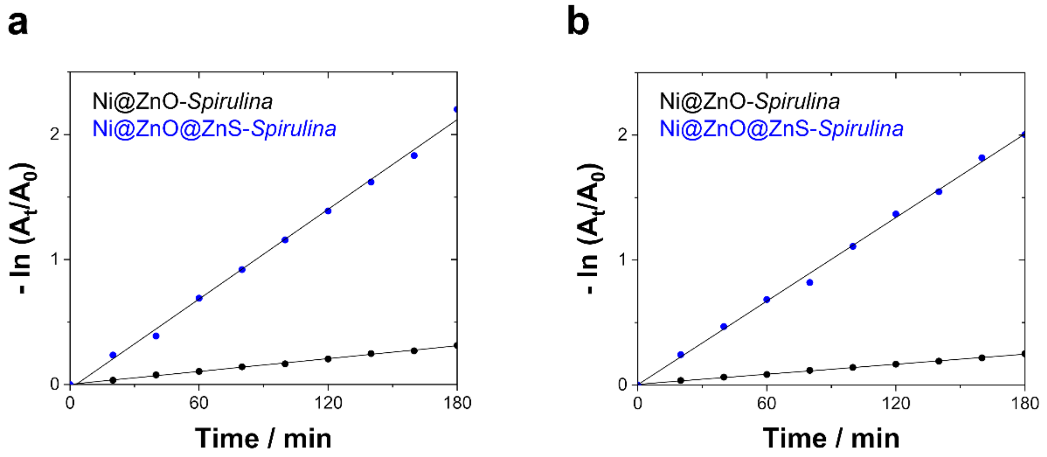

Figure S10: Kinetic linear simulation curves of methylene blue photodegradation under UV-filtered (λ > 400 nm) (a) artificial and (b) natural sunlight (photocatalyst dosage: 0.5 mg mL^{-1}).

11. Kinetic performance of the ZnO-based photocatalysts for the remediation of Methylene Blue

Table S2: Comparison of the photocatalytic performance of different ZnO-based photocatalyts previously reported in literature for the mineralization of methylene blue (25°C)

Photocatalyst	Light source	Methylene blue concentration [ppm]	m_{cat} [mg mL^{-1}]	k_{app} [min^{-1}]	k_{nor} [min^{-1} g^{-1}]	Reference
Ni@ZnO@ZnS-Spirulina	Visible	10	0.5	0.0163	8.0	This work
ZnO@N-C hybrid composite	UV	10	0.4	0.0456	2.28	1
ZnO/C	Visible	13	0.19	0.0300	2.0	2
ZnO@ZnS micro/nanoferns	Visible	10	0.4	0.0662	8.28	3
ZnO@ZnS NWs	Visible	20	-	0.0419	-	4
Sn:Cu:ZnO NPs	UV	20	0.001	0.0103	0.10	5
$Zn_3(VO_4)_2/Zn_2V_2O_7/ZnO$	Visible	10	0.6	0.0421	1.40	6

12. Photolysis of Methylene Blue

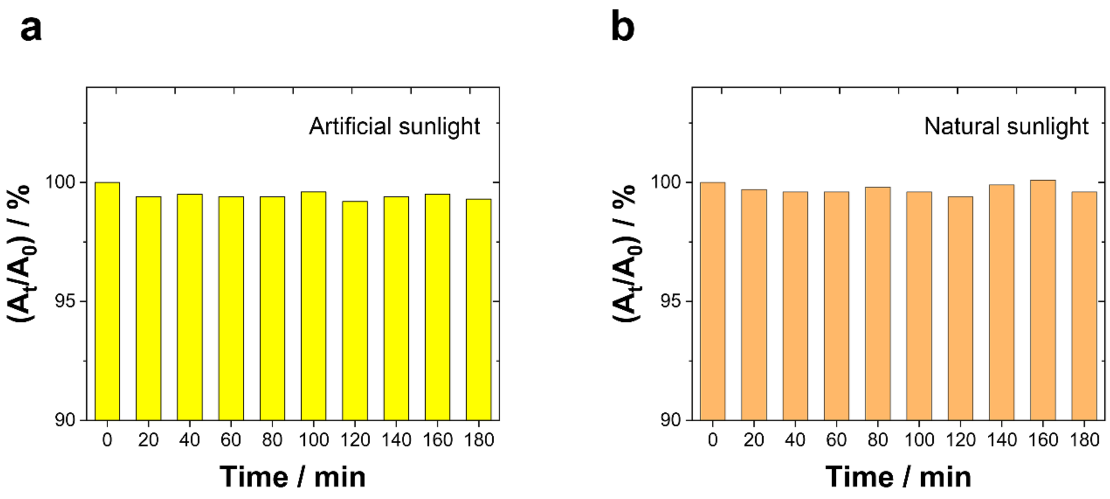

Figure S11: Time-dependent maximum absorbance of 10 ppm methylene blue under UV-filtered ($\lambda > 400$ nm) (a) artificial and (b) natural sunlight irradiation.

13. Trapping experiments

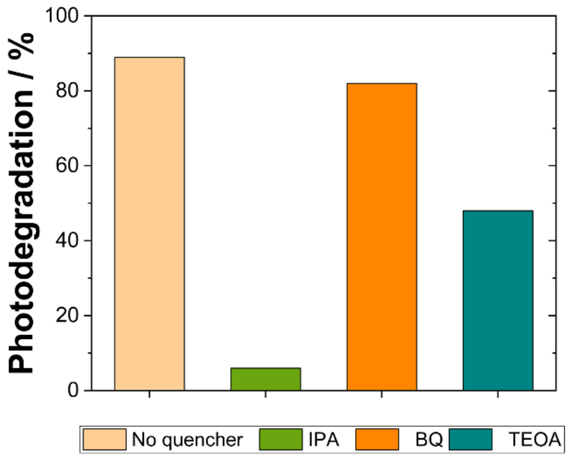

Figure S12: Trapping experiments of reactive species during the MB photodegradation using Ni@ZnO@ZnS-S*pirulina*. IPA, BQ, and TEOA are used to indicate isopropyl alcohol, benzoquinone, and triethanolamine quenchers, respectively. Photocatalyst dosage = 0.5 mg mL^{-1}; temperature = 25 ± 0.2°C; Irradiation: UV-filtered simulated sunlight (> 400 nm, light intensity of 680 ± 10 lx); irradiation time = 180 min.

14. FE-SEM micrographs of reused Ni@ZnO@ZnS-S*pirulina*

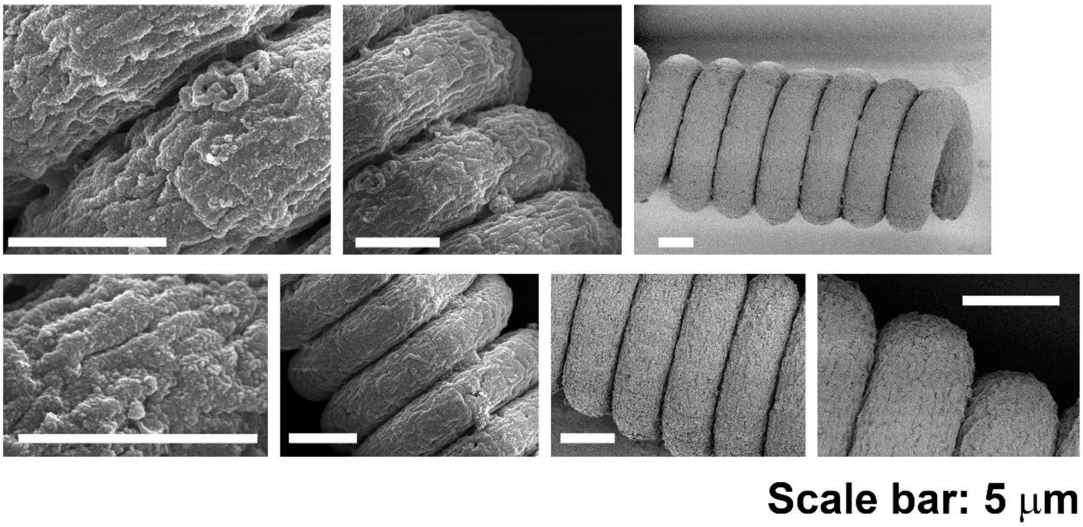

Figure S13: FE-SEM micrographs of Ni@ZnO@ZnS-S*pirulina* after being reused 25 cycles.

15. Enzymatic saccharification

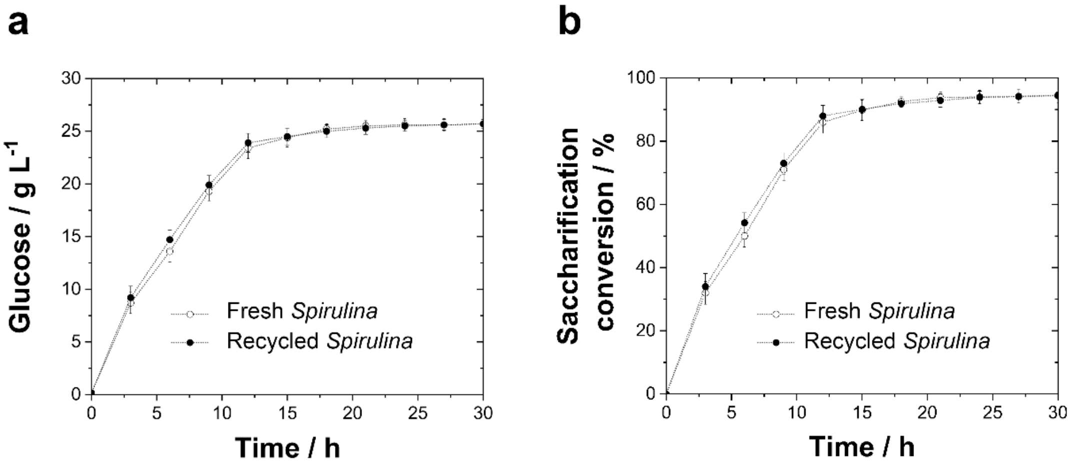

Figure S14: (a) Glucose production and (b) enzymatic saccharification conversion from fresh *Spirulina* (open circles) and Ni@ZnO@ZnS-S*pirulina* (close circles) in the presence of 1.5 U L^{-1} α-Glucosidase from *Saccharomyces cerevisiae* and 3.5 U L^{-1} α-Amylase from porcine pancreas at 31°C. Data points are mean values from three separate cultures with their corresponding standard deviation.

16. Ethanol yield from various bioethanol sources

Table S3: Comparison of the ethanol yield production for various bioethanol sources

Bioethanol source	Ethanol yield [mg g^{-1}]	Ethanol yield [L kg^{-1}]	Reference
Recycled *Spirulina*	323	0.41	This work
Spirulina microalgae	350	0.44	7
Barley	320	0.41	7
Corn	360	0.46	7
Oat	320	0.41	7
Rice	380	0.48	7
Chlorella	160	0.21	8
Rice straw	32	0.04	9
Pomegranate peels	80	0.10	10
Monterey pine slurry	205	0.26	11
Rice straw	210	0.27	12
Oil palm	450	0.57	13
Sweet potato	130	0.16	14

References

[1] R. Atchudan, T. N. J. I. Edison, S. Perumal, N. Karthik, D. Karthikeyan, M. Shanmugam, Y. R. Lee, *J. Photochem. Photobiol. A Chem.* **2018**, *350*, 75.

[2] H. Wang, X. Liu, S. Wang, L. Li, *Appl. Catal. B Environ.* **2018**, *222*, 209.

[3] A. Serrà, Y. Zhang, B. Sepúlveda, E. Gómez, J. Nogués, J. Michler, L. Philippe, *Appl. Catal. B Environ.* **2019**, *248*, 129.

[4] K. S. Ranjith, R. B. Castillo, M. Sillanpaa, R. T. Rajendra Kumar, *Appl. Catal. B Environ.* **2018**, *237*, 128.

[5] V. Shanmugam, K. S. Jeyaperumal, *Appl. Surf. Sci.* **2018**, *449*, 617.

[6] D. Zeng, K. Yang, C. Yu, F. Chen, X. X. Li, Z. Wu, H. Liu, *Appl. Catal. B Environ.* **2018**, *237*, 449.

[7] S. Aikawa, A. Joseph, R. Yamada, Y. Izumi, T. Yamagishi, F. Matsuda, H. Kawai, J. S. Chang, T. Hasunuma, A. Kondo, *Energy Environ. Sci.* **2013**, *6*, 1844.

[8] Y. K. Oh, K. R. Hwang, C. Kim, J. R. Kim, J. S. Lee, *Bioresour. Technol.* **2018**, *257*, 320.

[9] M. A. Ahmed, M. S. U. Rehman, R. Terán-Hilares, S. Khalid, J. I. Han, *Energy Convers. Manag.* **2017**, *141*, 120

[10] S. Talekar, A. F. Patti, R. Vijayraghavan, A. Arora, *Bioresour. Technol.* **2018**, *266*, 322.

[11] C. Dong, Y. Wang, H. Zhang, S. Y. Leu, *Bioresour. Technol.* **2018**, *250*, 102.

[12] Y. Li, P. Liu, J. Huang, R. Zhang, Z. Hu, S. Feng, Y. Wang, L. Wang, T. Xia, L. Peng, *Green Chem.* **2018**, *20*, 2047.

[13] E. Derman, R. Abdulla, H. Marbawi, M. K. Sabullah, *Renew. Energy* **2018**, *129*, 285.

[14] J. O. Virgínio e Silva, M. F. Almeida, M. da Conceição Alvim-Ferraz, J. M. Dias,

Renew. Energy **2018**, *124*, 114.
